# Effect of ovarian cancer ascites on SKOV-3 cells proteome: new proteins associated with aggressive phenotype in epithelial ovarian cancer

**DOI:** 10.1186/s12953-018-0133-9

**Published:** 2018-02-13

**Authors:** Alfredo Toledo-Leyva, Julio César Villegas-Pineda, Sergio Encarnación-Guevara, Dolores Gallardo-Rincón, Patricia Talamás-Rohana

**Affiliations:** 1 Centro de Investigación y de Estudios Avanzados del Instituto Politécnico Nacional, Av. Instituto Politécnico Nacional 2508, Col. San Pedro Zacatenco, Delegación Gustavo A. Madero, 07360 Ciudad de México, Mexico; 2 Centro de Ciencias Genómicas, Universidad Nacional Autónoma de México, Av. Universidad s/n Col. Chamilpa, 62210 Cuernavaca, Morelos Mexico; 3 Instituto Nacional de Cancerología, Av. San Fernando No. 22, Col. Sección XVI Delegación Tlalpan, 14080 Ciudad de México, Mexico; 4 Present address: Centro de Investigación de Cáncer en Sonora, Ciudad Obregón, Sonora 85010 Mexico

**Keywords:** Proteomics, Malignant cells, Ascites, Epithelial ovarian cancer, Aggressive phenotype

## Abstract

**Background:**

Epithelial ovarian cancer is the second most lethal gynecological cancer worldwide. Ascites can be found in all clinical stages, however in advanced disease stages IIIC and IV it is more frequent and could be massive, associated with worse prognosis. Due to the above, it was our interest to understanding how the ascites of ovarian cancer patients induces the mechanisms by which the cells present in it acquire a more aggressive phenotype and to know new proteins associated to this process.

**Methods:**

A proteomic analysis of SKOV-3 cells treated with five different EOC ascites was performed by two-dimensional electrophoresis coupled to MALDI-TOF. The level of expression of the proteins of interest was validated by RT-PCR because several of these proteins have only been reported at the messenger level.

**Results:**

Among the proteins identified that increased their expression in ascites-treated SKOV-3 cells, were Ran GTPase, ZNF268, and Synaptotagmin like-3. On the other hand, proteins that were negatively regulated by ascites were HLA-I, HSPB1, ARF1, Synaptotagmin 1, and hnRNPH1, among others. Furthermore, an interactome for every one of these proteins was done in order to identify biological processes, molecular actions, and cellular components in which they may participate.

**Conclusions:**

Identified proteins participate in cellular processes highly relevant to the aggressive phenotype such as nuclear transport, regulation of gene expression, vesicular trafficking, evasion of the immune response, invasion, metastasis, and in resistance to chemotherapy. These proteins may represent a source of information which has the potential to be evaluated for the design of therapies directed against these malignant cells that reside on ovarian cancer ascites.

## Background

Epithelial ovarian cancer (EOC), a multifactorial disease of unknown origin, is the second most lethal gynecological cancer worldwide. Mutations in genes become a 10% risk of presenting EOC and women over 50 years have an 80% risk of developing the disease [1, 2]. The International Federation of Gynecology and Obstetrics has established several clinical stages (CS); in stage IIIC and IV, usually an accumulation of ascites in the abdominopelvic cavity occurs [3]. This accumulation happens because malignant cells with an increased rate of proliferation and invasion reach lymph vessels blocking the draining process, thus, triggering a hydrodynamic imbalance in the peritoneal cavity causing active accumulation of fluid [4, 5]. Accumulation of ascites occurs in about 30 to 35% of patients and in all histological subtypes of EOC, especially in CS IIIC and IV [6]. Once established in the abdominal cavity, cells can carry on transcoelomic metastasis, a type of continuity metastasis that allow them to invade the abdominopelvic cavity, forming micro-implants in different organs. This fluid stimulates a more aggressive cellular phenotype complicating the clinical prognosis [6–9]. Puiffe et al., (2007) analyzed 54 independent samples of ascites and determined the effect of this fluid in the invasive phenotype of EOC cell lines; they found that a good percentage of these ascites induced invasiveness of these cells, while a good proportion of the fluids produced the opposite effect. Finally, a small number apparently resulted irrelevant in this stimulating effect. This demonstrates that each ascites is different and complex, even when dealing with the same disease [6].

The immune response is actively involved in the progression of this disease; paradoxically, to eliminate malignant cells, immune cells induce inflammation, a process that is surpassed by malignant cells as they learn to respond to several factors such as chemokines and growth factors [10]. The composition of this fluid will largely depend on the body’s response to the neoplastic process and the molecules used by these cells in complex mechanisms to avoid their elimination. Ascites contains molecules as bioactive lipids, nutrients, glycoproteins, and cytokines among others, that modulate different cellular functions and thus cell behavior [11, 12]. Malignant cells may use different strategies that allow them to achieve disease progression, such as evasion of the immune response, negative regulation of apoptosis, increased proliferation and migration along with the development of distant metastasis sites [13, 14]. Moreover, several pro and anti-inflammatory cytokines, growth factors, and proteases have been identified in ascites that help these cells to carry out the afore-mentioned processes.

Among the main characteristics of these cells are, a higher rate of migration and their ability to establish a very dynamic relationship with the tumor microenvironment [8, 15]. Thus, it is highly relevant to recognize the effect that the tumor microenvironment, represented in the ascites of EOC patients, may exert on malignant cells, producing phenotypic changes that defines their behavior and thus the prognosis for each patient [6].

In this work, we performed a proteomic analysis of SKOV-3 cells under ascites condition that allowed us to identify, for the first time in this disease, several proteins associated with different malignant processes. Among the identified proteins were Ran GTPase, Zinc finger protein 268, and synaptotagmin like-3, human leucocytic antigen-I, heat shock protein beta-1, ARF1, SYT1, and hnRNPH1. These proteins participate in important cellular processes highly relevant to the aggressive phenotype such as nuclear transport, regulation of gene expression, vesicular trafficking, evasion of the immune response, invasion, metastasis, and in resistance to chemotherapy.

## Methods

### Treatment of SKOV-3 cell line with EOC ascites

Originally, five different ascites were selected for SKOV-3 cells treatment. Due to differences in the collected volumes of these samples, inherent to the health status of the patients, only four ascites were used for the validation experiments. Previous to cells treatment, ascites were defrosted and centrifuged at 18,620 x *g*, 10 min at 4 °C and then warmed at 37 °C. Cells (3.5 × 10^5^) were incubated individually for 72 h with 15 ml of each of the five different ascites: A1, A2, A3, A4, and A5 (Table 1); cells in complete McCoy’s culture medium, were used as reference for comparisons.

**Table 1:** Clinical characteristics of ascites from EOC patients

"*"MRN	Ascites No.	Age	Histological Subtype	Clinical Stage	CA125
43,476	A1	52	**HG Serous papillary	IIIC	25,772
113,929	A2	57	Metastatic from mama cancer	IIB	1628
120,200	A3	39	HG Serous papillary	IVA	12,300
113,889	A4	56	Mucinous	IIIC	467
124,556	A5	70	HG Serous papillary	IVA	132

*MRN: Medical record number; ** HG, High grade

### Two-dimensional electrophoresis (2-DE) assays

At the end of the 72 h incubation, cultured cells were detached using 0.01% trypsin/0.1 M EDTA and the reaction was stopped by adding culture medium supplemented with 10% FBS. Cells were then centrifuged at 139 *x g* for 10 min at 4 °C, quickly rinsed and washed with sterile 1× PBS twice; finally, cells were centrifuged at 9500 *x g* for 10 min at 4 °C. The cell pellet was subjected to lysis as described above and protein quantification was performed using the Bradford method [16]. Total protein extracts (500 μg) were cleaned of salts and detergents using the phenolic extraction protocol as follows: first, proteins were precipitated with cold acetone for 16 h at − 20 °C. Pellet was then resuspended in extraction buffer consisting of 0.5 M Tris-HCl, 0.7 M sucrose, 30 mM HCl, 50 mM EDTA, 0.1 M KCl, 12 mg/ml polyvinylpolypyrrolidone and 2% mercaptoethanol. A comparable volume of saturated phenol was added to each sample and then they were centrifuged at 17,780 x *g* for 10 min. The aqueous phase was then precipitated with 5 volumes of 0.1 M ammonium acetate in methanol at − 20 °C overnight. Precipitates were washed with 0.1 M ammonium acetate and 80% acetone and dried under vacuum. Finally, cleaned proteins were resuspended in lysis buffer consisting of 8 M urea, 2 M thiourea, 4% CHAPS, 2% ampholines (pH range 4–6.5, 5–8, and 3–10) and 60 mM dithiothreitol.

Proteomic profiles (350 μg) of the five experimental and one control condition were performed. Isoelectric focusing was performed using 19 cm strips with a pH gradient of 3–10 (GE Healthcare Pharmalyte TM, Cat. No.: 170,456–01), a pH range of 4–6.5 (GE Healthcare Pharmalyte TM, Cat. No.: 17–0452-01), and a pH range of 5–8 (GE Healthcare Pharmalyte TM, Cat. No.: 17–0453-01). The isoelectrofocusing was performed for 24 h at 22 °C. For the second dimension, strips were mounted on a 19 × 23 cm 12.5% SDS-PAGE, run at 250 V for 24 h at 22 °C. Three replicates for each condition (one control and five experimental conditions) were performed; 18 gels in total for this study. Finally, gels were fixed and stained with colloidal Coomassie blue. Gels were neutralized to remove the background generated by the dye; then, gels were washed with 25% methanol in 0.1 M Tris-HCl pH 6.5. Finally, they were placed in 20% ammonium sulfate and were digitized for proteomics maps using a densitometer (Bio-Rad Hercules, GS-800, CA, USA) [17]. Gels were fixed and stained with colloidal Coomassie blue G-250, and scanned with PDI image analysis system (Bio-Rad Hercules, GS-800, CA, USA), to obtain the proteomic profiles.

### Bioinformatics analyses of proteomics profiles

Proteomics profiles were analyzed using the 8.0.1 PDQuest software version [18, 19]. Protein spots were detected automatically by the software in each of the replicas and later manual adjustment and editing of maps was carried out to remove artifacts erroneously detected as protein spots or to add those undetected. Subsequently, proteomic profiles were matched with their corresponding spots in each of the biological replicates. Those protein spots that showed an up or down change in their expression by 2-fold or more were chosen for further analysis by mass spectrometry.

### Mass spectrometry (MALDI-TOF) and protein identification

These procedures were performed as previously described [17]. Briefly, selected spots from Coomassie stained 2-DE gels were excised manually and then proteins were reduced with 60 mM dithiothreitol, alkylated with 40 mM iodoacetamide, and digested for 24 h at 37 °C, with sequencing degree modified trypsin (Cat. No. V5111, Promega) for the generation of peptides. Then, peptides were recovered using a matrix and placed on a steel plate and introduced into the MALDI-TOF equipment (Matrix-Assisted Laser Desorption/Ionization-Time of Flight, Autoflex Brunker Daltonics Billerica, MA, USA). The Proteinner SP and SPII systems (Bruker Daltonics, Breme, Germany SPcontrol 3.1.48.0v software) were used. The Bruker Daltonics Autoflex system was configured in delayed extraction and in reflectron mode. The *m/z* values obtained were compared against protein sequences of *Homo sapiens* databases in NCBInr and SwissProt using Mascot 2.0 as a search engine, with the following parameters: a cleavage site lost, carbamidomethylation cysteine as fixed modification, and methionine oxidation as a variable modification. Proteins with a score higher than 67 and a threshold of significance of *p* < 0.05 were accepted as positive identifications. However, we did include in Table 1, two additional proteins with a 64 score because they were outside the shadow zone; this is the case of PDZD11 and SYTL3 proteins, whose identity should be taken with caution.

### RT-PCR assays

To confirm the expression level of the identified proteins that were down or up regulated by effect of EOC ascites, RT-PCR assays were performed [20, 21]. First, total mRNA was obtained from whole extracts of SKOV-3 and SKOV-3 cells using TRIZoL reagent (Cat. No. 10296028, Thermo Fisher Scientific) following the manufacturers’ protocol. Synthesis of cDNA was carried out from this mRNA using Single Chain Synthesis Kit (Thermo Fisher Scientific Cat. No. K1612). Design of primers was made using PRIMER3 [22] and Primer BLAST-NCBI [23] Primers used for these assays were from Sigma Aldrich and the specific sequences were the following: RAN, forward agagccccaggtccagttcaaa, reverse cccaaggtggctacatacttct; SYTL3, forward aagcgcctgttcaactttgtc, reverse aggttggaagagcttcactgc; ZNF268, forward gcgagatccttgttcctcag, reverse cctgaccttggagctttctg, HLA-I, forward ctgtggtggtgccttctgg, reverse cacaactgctaggacagcca; SYT1, forward tccaggccacaagacagtag, reverse agcctaccatcagccctttt; ARF1, forward accccgcctagcatagattt, reverse cacatggctatggaatgcag; HSPB1, forward acgagcatggctacatctcc, reverse ctttacttggcggcagtctc; hnRNPH1, forward gtgcagtttgcttcacagga, reverse ccccaggtctgtcataagga. GAPDH was used as a housekeeping gene and results were normalized against this internal control. The amplification was performed using PCR Master Mix (Thermo Fisher Scientific Cat. No. K0171).

### Bioinformatic analyses of identified proteins

Bioinformatic analyses were performed using the Gene Ontology database using Gene Ontology Terms (GOTERM); DAVID and STRING 10.0 for functional groupings and for protein-protein interaction (PPI) networks to determine the interactions in which they might be participating under our experimental conditions.

### Statistical analyses

For each of the experimental procedures followed, specific statistical analyses were applied. To determine the expression level of proteins on the 2-DE analyses, a normalization of proteomic maps was developed using local regression model with the PDQuest ver. 8.0.1 software provided by BioRad Laboratories. This allowed the comparison of each of the replicas of the control condition against replicas of the experimental conditions. Using a bioinformatic tool for quantitative comparison provided by the software, all protein spots represented in the samples with 95% statistical confidence (*p* < 0.01) in a Student’s t test were analyzed.

To analyze the expression at the transcript level of deregulated genes, a densitometry analysis was performed from RT-PCR results, where at least three biological replicas for each gene were done; GAPDH was used as a housekeeping gene. ANOVA test was performed to determine changes between each ascites compared against control condition, and Bonferroni post hoc for this group of data, * = *p* < 0.05, ** = *p* < 0.001, *** = *p* < 0.0001.

## Results

**Fig. 1:**
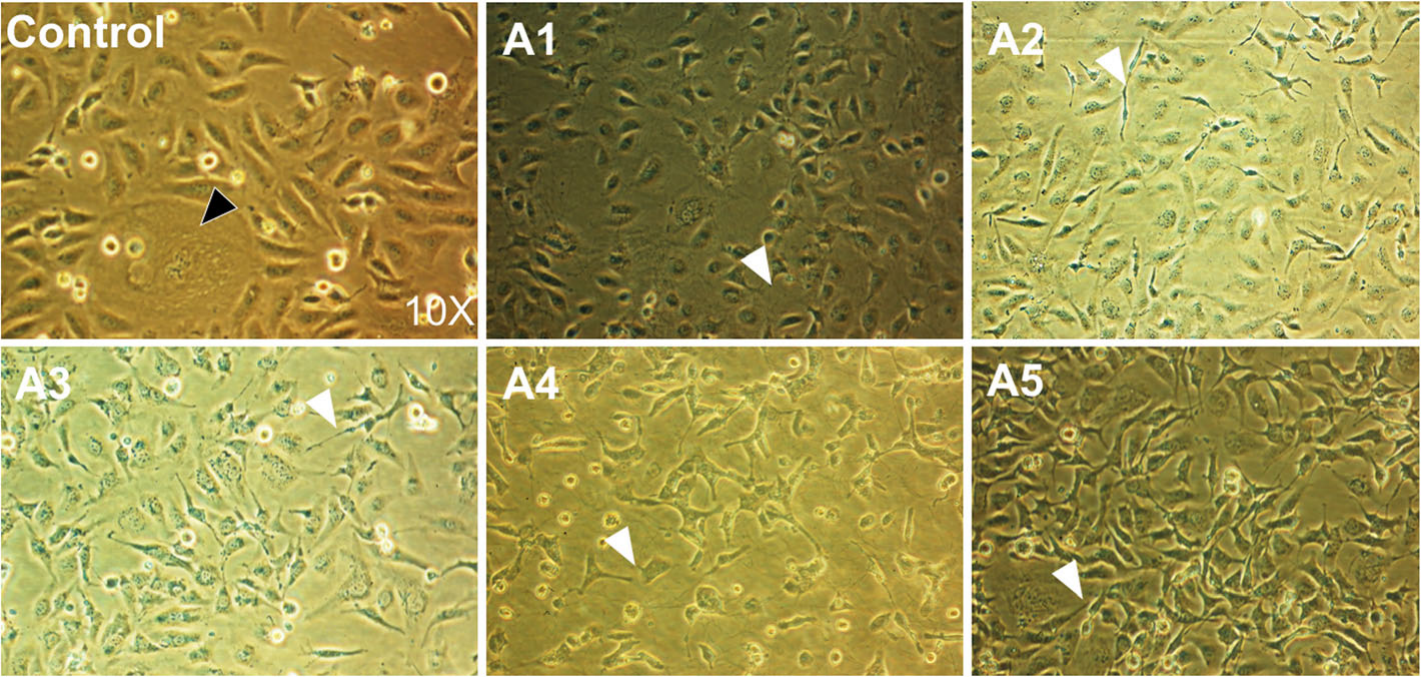
Treatment of SKOV-3 cells with ascites from EOC patients. Cultures of SKOV-3 cell line were treated individually with five different ascites; Control, cells incubated in conventional culture medium with 10% of SFB used as reference for comparisons; A1 - A5, correspond to the five tested ascites. Black arrowhead indicates the presence of a large round cell with abundant cytoplasm; white arrowheads indicate membrane protrusions such as invadopodia, lamelipodia and filopodia present in cells treated with EOC ascites. General conditions of incubation for control and treatments were 72 h at 37 °C and 5 %CO_2_

**Fig. 2:**
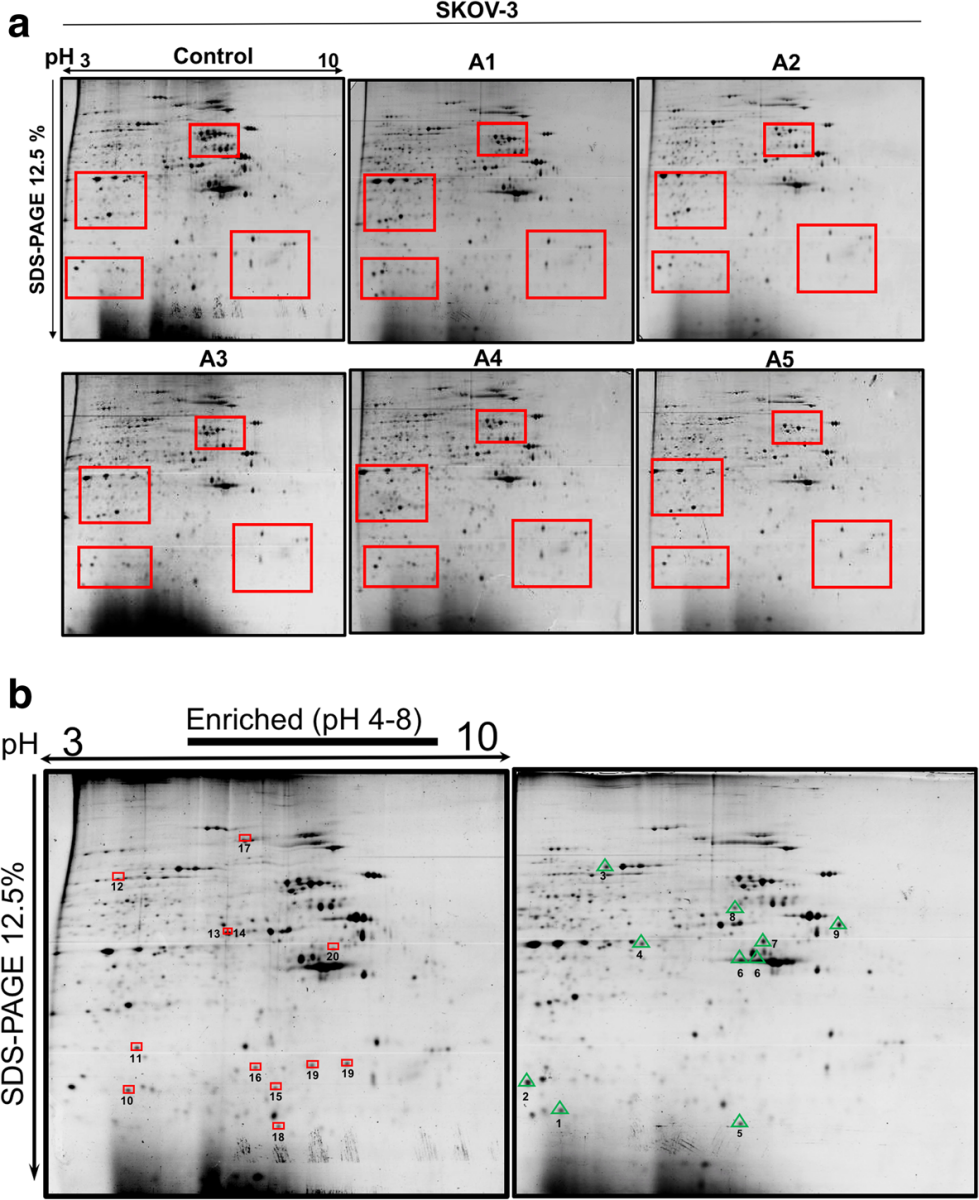
2-DE patterns of protein extracts from SKOV-3 cells, non-treated (control) or treated with five different ascites (A1-A5). **b**. 2-DE gels showing the definition of at least 300 proteins spots. Regions with differential protein expression patterns were identified (red squares). **b**. Analyses of proteomic maps using the PDQuest software Ver. 8.0.1. Proteins with a two-fold down-regulated expression (11, red squares, left panel) and proteins with at least two-fold up-regulated expression (9, green triangles, right panel,) are shown. Gels were prepared in a large format at 12.5%; a pH gradient from 3 to 10, with an enrichment from 4 to 8 pH was applied

Ascites generated in advanced stages of EOC form an important tumoral dynamic microenvironment that modulate cells’ behavior, composition and morphology. [24–26]. Thus, ascites was used to treat SKOV-3 cells to analyze their impact at the proteomic level. Figure 1 shows the effect of the different ascites on the cellular morphology, confirming that indeed these ascites were affecting the cells. As can be seen, most of the cells incubated in EOC ascites reduced their size and formed great membrane protrusions such as invadopodia, lamelliopodia and filopodia (Fig. 1, A1-A5, white arrowheads). Whereas in culture medium large round cells can be found (Fig. 1, control, black arrowhead), and most of the cells are bigger in size. Once we confirmed by cell morphology that the ascites was modifying SKOV-3 cells, we proceeded with their proteomic analysis. Total extract of proteins from ascites-treated SKOV-3, as well as from cells incubated in culture medium were analyzed by 2-DE gel electrophoresis to obtain a proteomic profile in both conditions. Around 300 spots were detected in each of the gels analyzed; at least four areas of differential expression between each of the proteomic profiles were detected, both for the control condition and for the experimental treatments (Fig. 2a, red squares). Differences between the control and experimental conditions were not highly pronounced but the densitometry analyses allowed to detect at least 20 spots that showed alterations in their expression. Among them, 9 proteins showed an increased level of expression (Fig 2b, green triangles in a representative gel of SKOV-3 cells incubated with ascites), whereas 11 showed a decrease in their expression levels (Fig. 2b, red boxes in the gel of SKOV-3 cell line under culture medium condition), in either case in at least two times or more (Table 2). Proteins that were exclusively expressed in any of the conditions were selected for further characterization.

**Table 2:** Deregulated proteins identified by MALDI-TOF in SKOV-3 cells by effect of EOC ascites (*n* = 5)

Spot No.		Protein Name	Gene Name	Uniprot ID	NCBI/ SwissProt Accession #	Mw (kDa)	Sequences Matched	Mascot Score	Regulation (Folds)	SD
Upregulated proteins	1	PDZ domain containing protein 11	PDZD11	Q5EBL8	PDZ11_HUMAN	16.1	7	64	+ 2.5	1.5
	2	GTP-binding nuclear protein Ran	RAN	P62826	gi|48,734,884	24.6	9	90	+ 2.8	0.51
	3	Zinc finger protein 268	ZNF268	Q14587	gi|14,579,579	92.6	11	68	+ 2.5	0.62
	4	Succinyl-CoA:3-ketoacid-coenzyme A transferase1, OXCT1	N/A	B7Z609	gi|363,981,034	36.8	5	72	+ 3.2	1.2
	5	Collagen alpha 6(VI) chain	COL6A6	A6NMZ7	gi|767,925,744	183.4	10	68	+ 2.8	0.05
	6	Actin B	ACTB	P60709	gi|15,277,503	40.5	9	103	+ 2.4	0.78
	7	Keratin, type II cytoskeletal 8	KRT8	P05787	gi|119,617,058	53.4	11	75	+ 2.3	0.83
	8	Synaptotagmin-like protein 3	SYTL3	Q4VX76	SYTL3_HUMAN	70	8	64	+ 2.5	0.52
	9	Vimentin	VIM	P08670	gi|47,115,317	53.6	9	79	+ 2.7	0.12
Downregulated proteins	10	Human Leucocytic Antigen I	HLA-I	P01892	gi|326,416,438	21.7	6	79	- 2.3	0.11
	11	Purine nucleoside phosphorylase	PNP	P00491	gi|37,926,571	32.2	7	69	- 2.1	0.38
	12	Prelamin-A/C	LMNA	P02545	gi|767,909,266	61.6	9	69	- 2.4	0.76
	13	Actin, cytoplasmic 2	ACTG1	P63261	gi|4,501,887	42.1	13	97	- 2.2	0.99
	14	Heterogeneous nuclear ribonucleoprotein H1	HNRNPH1	P31943	gi|767,938,360	50.5	7	75	- 2.5	1.0
	15	Heat shock protein beta-1	HSPB1	P04792	gi|662,841	22.4	7	91	- 2.9	0.88
	16	ADP ribosylation factor	ARF1	P84077	gi|545,719,724	44.6	8	69	- 2.4	0.09
	17	Lon protease mitochondrial	LONP1	P36776	gi|414,046	95.5	10	87	- 3.0	1.5
	18	Peroxiredoxin-2	PRDX2	P32119	gi|32,189,392	18.4	5	85	- 2.6	0.48
	19	Cathepsin D	CTSD	P07339	gi|672,886,498	26.7	6	77	- 2.5	0.89
	20	Synaptotagmin-1	SYT1	P21579	gi|167,744,962	32.5	8	68	- 2.5	1.2

After excision of spots and processing by MALDI-TOF peptide masses were obtained by peptide mass fingerprinting from the ionized peptides. Table 2 shows the proteins identified.

**Fig. 3:**
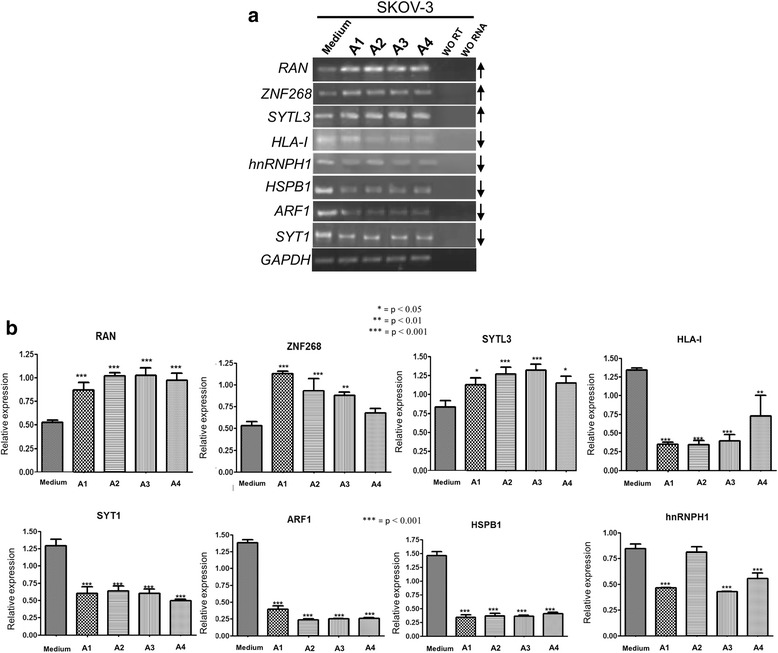
mRNA expression level of proteins identified to be modified in SKOV-3 cells. **a**. mRNA expression profiles of three upregulated and five downregulated proteins in SKOV-3 cells. **b**. Densitometric analysis of selected up and downregulated genes at transcriptional level. At least three replicas were performed for each gene; GAPDH was used as a housekeeping gene. * = *p* < 0.05, ** = *p* < 0.001, *** = *p* < 0.0001

Eight proteins (three up- and five down-) regulated under the effect of ascites were selected to confirm their changes at the transcriptional level. Using 4 different ascites the expression level of RAN, ZNF268, SYTL3, HLA-I, hnRNPH1, HSPβ1, ARF1 and SYT1 was evaluated. After the induction of SKOV-3 cells with ascites, RAN, ZN268 and SYTL3 proteins increased twice their expression levels in some cases. On the other hand, HLA-I, hnRNPH1, HSPβ1, ARF1 and SYT1 proteins decreased two or three times their expression level (Fig. 3a). These results strongly support the results obtained by proteomic analysis of SKOV-3 cells under EOC ascites condition.

The statistics of the densitometry analyses of this set of data confirmed that the differences observed in ascites-treated SKOV-3 cells were significant compared against control condition (Fig. 3b) (* = *p* < 0.05, ** = *p* < 0.01, *** = *p* < 0.001).

**Fig. 4:**
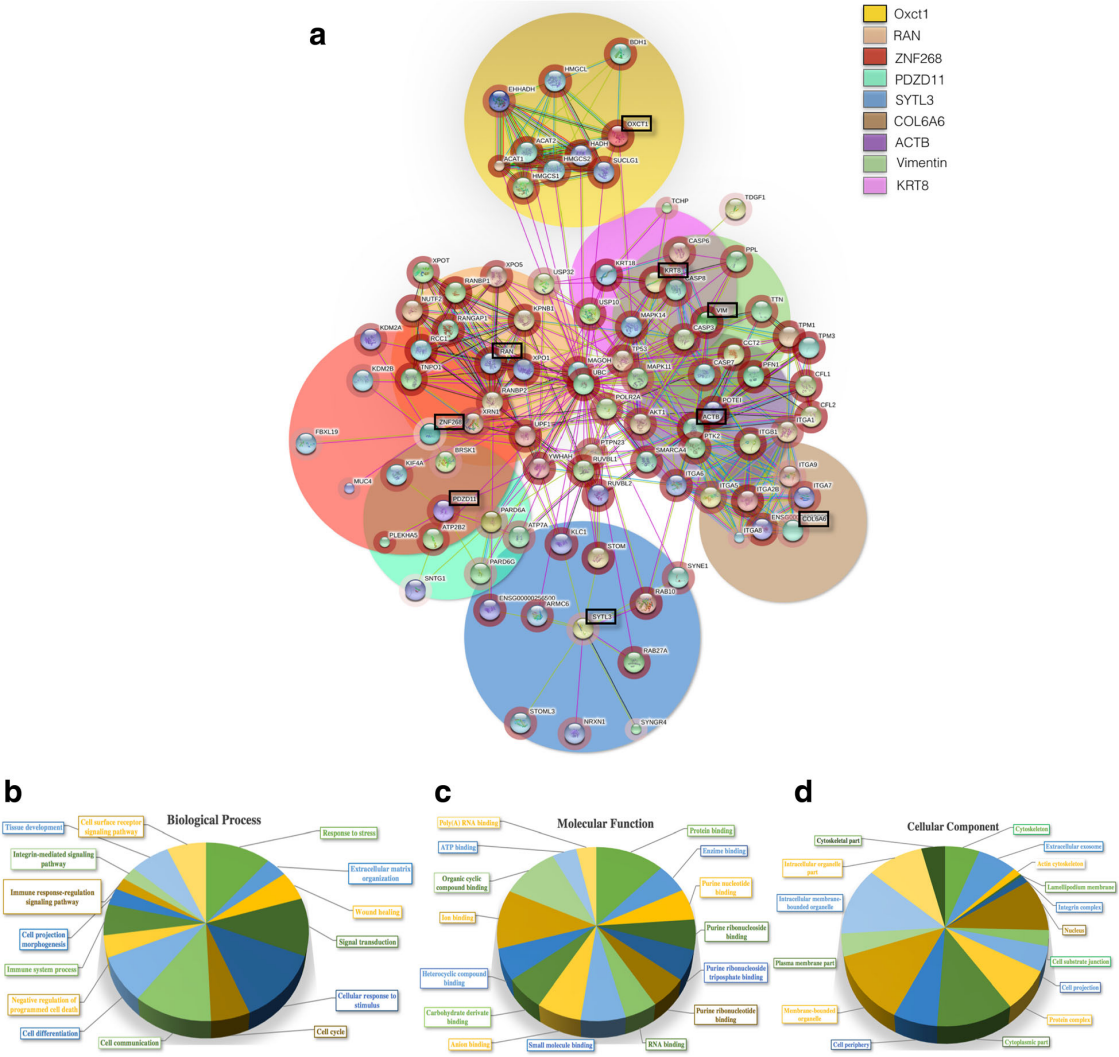
Protein-protein interaction (PPI) network and ontology analyses of upregulated proteins in SKOV-3 cells. **a**. PPI network was built using STRING Ver. 10.0 to analyze the nine increased proteins (indicated in black boxes) by effect of ascites; color circles indicate the main group of proteins related with these proteins at several biological and experimental levels. The intensity for the red halo around each protein indicates the relative abundance obtained from Pax DB for *Homo sapiens* proteins. Gene Ontology Terms (GOTERM) and annotations for the group of proteins upregulated and their main proteins interacting; gene ontology annotations were performed for a functional enrichment of Biological Process (**b**), Molecular Function (**c**) and Cellular Component (**d**) categories. The groups with a higher quantity of genes related to a specific action on each category were used as a representative action, function or localization under our experimental conditions

Once the deregulated proteins were identified, and their level of over- or down-expression was confirmed, we proceeded with the bioinformatic analyses using Gene Ontology, and Panther DB databases [27], and STRING 10 and Pax DB, [27, 28]. These analyses provide a view of the proteins that could possibly be interacting with the identified proteins (Fig. 4a, black boxes), to detect the biological processes that might be affected by the regulation of the identified proteins as a consequence of EOC ascites treatment. The PPI network shows the main group of proteins (indicated by the big color circles) with which these proteins interact. Also, the relative abundance of these proteins at the physiologic level is shown in the red halo surrounding each protein (Fig. 4a). Using the Gene Ontology (GO) enrichment analysis the identified up- and down-regulated proteins were classified in biological processes, molecular functions, and cellular components according to its typical function, using mainly DAVID database [29]. Upregulated proteins in SKOV-3 show a complex interrelation with other proteins in addition to its participation in processes closely related with a highly competent cellular phenotype to confront an adverse microenvironment. Their classification in biological processes (Fig. 4b), molecular functions (Fig. 4c), and as constituents of cell components (Fig. 4d) is shown in Table 3.

**Table 3:** Gene Ontology (GO) enrichment analysis to classify up-regulated proteins

UP-REGULATED PROTEINS	
A). BIOLOGICAL PROCESSES	GOTERM
Response to stress	0006950
Extracellular matrix organization	0030198
Wound healing	0042060
Signal transduction	0007165
Cellular response to stimulus	0051716
Cell cycle	0007049
Cell communication	0007154
Cell differentiation	0030154
Negative regulation of programmed cell death	0043069
Immune system process	0002376
Cell projection morphogenesis	0048858
Immune response-regulation signaling pathway	0002764
Integrin-mediated signaling pathway	0007229
Tissue development	0009888
Cell surface receptor signaling pathway	0007166
B). MOLECULAR FUNCTION	GOTERM
Protein binding	0005515
Enzyme binding	0019899
Purine nucleotide binding	00117976
Purine ribonucleoside triphosphate binding	0035639
Ion binding	0043167
Organic cyclic compound binding	0097159
C). CONSTITUENTS OF CELL COMPONENTS	GOTERM
Cytoskeleton	0005856
Extracellular exosome	0070062
Actin cytoskeleton	0015629
Lamellipodium membrane	0031258
Integrin complex	0008305
Nucleus	0005634
Cell substrate junction	0030055
Cell projection	0042995
Protein complex	0043234

**Fig. 5:**
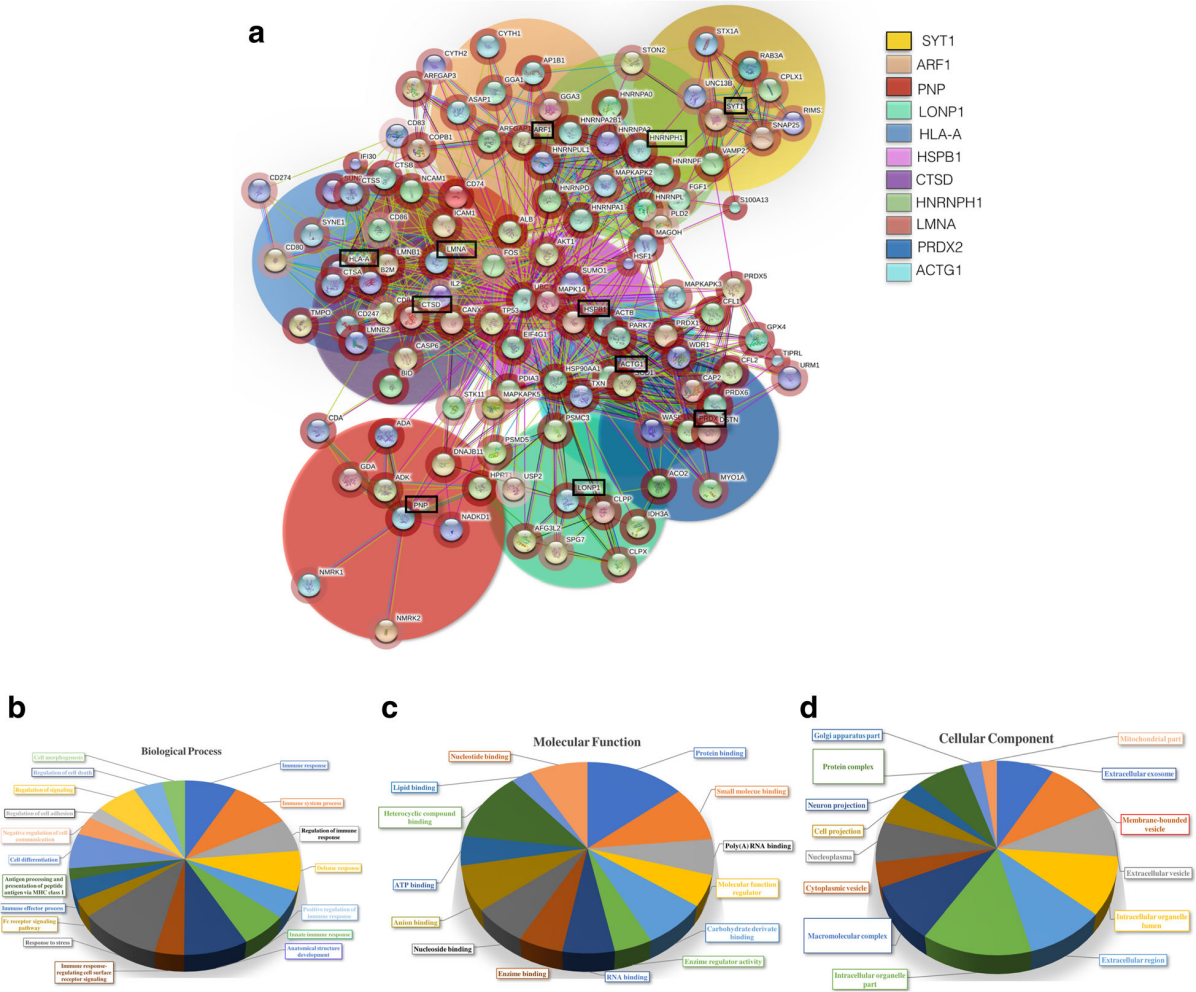
Protein-protein interaction (PPI) network and ontology analyses of downregulated proteins in SKOV-3. **a**. PPI network was built using STRING Ver. 10.0 to analyze the eleven decreased proteins (indicated in black boxes) by effect of ascites; color circles indicate the main group of proteins related with these proteins at several biological and experimental levels. The intensity for the red halo around each protein indicates the relative abundance obtained from Pax DB for *Homo sapiens* proteins*.* Gene Ontology Terms (GOTERM) and annotations for the group of proteins downregulated and their main proteins interacting; gene ontology annotations were performed for a functional enrichment of Biological Process (**b**), Molecular Function (**c**) and Cellular Component (**d**) categories. The groups with a higher quantity of genes related with a specific action on each category were used as a representative action, function or localization under our experimental conditions

The PPI network for downregulated proteins (Fig. 5a, black boxes) was performed using at least ten main interacting proteins contained within the big color circles for each identified protein. They were also classified as proteins associated with biological processes (Fig. 5b), with molecular functions (Fig. 5c), and as part of cellular components (Fig. 5d) (Table 4).

**Table 4:** Gene Ontology (GO) enrichment analysis to classify down-regulated proteins

DOWN-REGULATED PROTEINS	
A). BIOLOGICAL PROCESSES	GOTERM
Immune response	0006955
Immune system process	0002376
Regulation of immune response	0050776
Defense response	006952
Positive regulation of immune response	0050778
Innate immune response	00450
Cell differentiation	003030154
Regulation of signaling	0023051
Cell morphogenesis	0000902
Negative regulation of cell communication	0010648
Response to stress	0006950
B). MOLECULAR FUNCTIONS	GOTERM
Enrichment for protein binding	0005515
Small molecule binding	0036094
Poli (A) RNA binding	0044822
Molecular regulator	0030234
Enzyme binding	00119899
C). CONSTITUENTS OF CELLULAR COMPONENTS	GOTERM
Extracellular exosomes	0070062
Membrane-bounded vesicle	0031988
Intracellular organelle lumen	0070013
Nucleoplasm	0005654
Golgi apparatus	0044431
Mitochondria	004429

## Discussion

A proteomic analysis was performed with the SKOV-3 cell line treated with different ascites from EOC patients, to identify proteins that were deregulated, thus reflecting the state of these cells. We recognize that the cell line SKOV-3 may not be the closest to a high-grade serous ovarian carcinoma; however, SKOV-3 cell line has been widely used for ovarian cancer studies (2101 citations in PubMed). We thus decided to use this line as the target of different ascites and this approach allowed us to identify new proteins important for the malignant transformation process. Definitively, as stated by the Expert Committee on the State of the Science in Ovarian Cancer Research (2016), “…the incomplete understanding of the basic biology of each subtype of ovarian carcinoma is an impediment to advances in prevention, screening and early detection, diagnosis, treatment, and supportive care” [30]. Therefore, for subsequent studies and in accordance with the recommendations issued by this expert committee it will be important to carry out research oriented to specific histological subtypes and thus avoid generalizations. Also, as stated in the summary of the manuscript by Domcke et al., (2013) “…the gap between cell lines and tumours can be bridged by genomically informed choices of cell line models for all tumour types [31]” which we will consider for future research.

Proteins with more than twofold average quantitative expressions between ascites and culture medium were considered as regulated proteins. Among them, eight were selected for further validation and discussion about their possible role in cancer development. The main reason why our results were validated by RT-PCR was because several of the proteins have only been reported at the messenger level; these proteins have been poorly studied at the level of expression of the protein. Such is the case for ZNF268, STYL3 and SYTL1 (in addition to others identified in our proteomic analysis). The validation and monitoring of some of these proteins of interest such as HLA-1, RAN and Vimentin are currently being studied by our research group and these results will be part of another experimental approach that will help us explain the aggressive phenotype of cells under this tumor microenvironment.

The upregulated proteins selected and confirmed at the transcriptional level were Ran, Zinc finger protein 268, and synaptotagmin like-3 proteins. Ran is an important small GTPase implicated in the nuclear transport of diverse proteins that participate in several processes and it has been reported that this protein is over-expressed in breast and renal cancer and has also been linked to the development of tumorigenesis and metastasis [32, 33]. Zinc finger protein 268 functions as a transcriptional repressor and has been associated with cervical-uterine cancer where it was found overexpressed, and associated with increased tumorigenesis; the knockdown of this protein showed that it relates to increased signaling of NF-κB, contributing to tumorigenesis, cell proliferation, and growth [34]. Finally, synaptotagmin like-3, is a protein that can function as an effector for Rab proteins involved in vesicular trafficking; it also binds to phospholipids in the presence of calcium in neuron cells, and some functions of this protein as the binding to several vesicles are inferred because its function isn’t well-known [35]. It is important to emphasize that this protein has not been previously associated with any type of cancer, which makes it a molecule with the potential to be evaluated as a possible biomarker [36, 37]. It’s also important to mention that vimentin, considered as a mesenchymal marker, was found upregulated by influence of the five EOC ascites tested in the proteomic analysis (Table 2).

Downregulated proteins selected to validate at the transcript level were associated mainly with processes such as transport, immune evasion, and stress response. HLA-I, is a protein found on the cell surface of all nucleated cells that functions as a complex for antigen presentation to cytotoxic T cells. In lung cancer, it has been suggested that the reduction of this protein is used as an escape mechanism from immune surveillance [38, 39], whereas in ovarian cancer malignant cells, the decrease of this molecule has been reported as a mechanism to avoid their elimination mediated by CD8+ T cells [40]. To date, the most studied immune escape mechanisms in epithelial ovarian cancer are those related to PD1-PDL1 and CTLA-4 [41–43] . Therefore, the existence of an additional evasion mechanism in these mesenchymal cells could be highly relevant for immunomodulation therapy.

Heterogeneous nuclear ribonucleoprotein 1, a component of nuclear ribonucleoprotein complexes, is involved in the biogenesis of mRNA, by alternative splicing of the apoptotic mediator Bcl-x [44]; in cancer, it has been found overexpressed, and highly phosphorylated phenotypes correlated with HER2 positive cancer [45]. For its part, HSPB1 protein also called HSP27, is a member of a family of small heat shock proteins of human, characterized by the presence of a highly conserved alpha domain. This protein has a structural modulating chaperone activity [46, 47]. It has been reported that over expression of this protein is related with a highly invasive and migration cell phenotype; the HSPB1 knockdown revealed the loss of these features as well as a decrease in the ability to develop metastases [48, 49]. In the case of Arf1, numerous studies suggest that its increased expression is linked to chemotherapy resistance. This protein is an ADP-ribosyl transferase involved in protein trafficking among different compartments, and that also regulates the formation of vesicles [50], functions relevant for proliferation, migration and differentiation.

On the other hand, synaptotagmin 1 can participate in the regulation of membrane interactions during synaptic vesicle trafficking in the dynamic zone of the synapse. Also, a calcium dependent interaction between synaptotagmin 1 and putative receptors for activated C kinase protein has been documented [51]. Moreover, there are reports about its participation in the formation of dendrites by melanocytes [51, 52]. However, how this protein functions within a specific process in cancer isn’t well-known.

Protein-protein interactions (PPIs) play essential roles in all biological processes. In vivo, PPIs occur dynamically and depend on the interaction between cells and their surroundings [53, 54]. Bioinformatic analyses using the complete list of proteins identified by proteomic analysis were used to perform the PPI networks [27]. These analyses allowed to recognize the impact of the changes induced by ascites on SKOV-3 cell line. The affected functions found using bioinformatic analysis tools are implicated in processes related with the induction of a very aggressive phenotype of these cells. The most remarkable processes are related with an increase of proliferation, migration, invasion, metastases and cell survival [55]. In the case of the upregulated proteins we found that proteins such as vimentin and keratin 8 are expressed in undifferentiated cells, which have a very high rate of migration and invasion; moreover, other proteins such as Ran help to transport several important factors into the nucleus and this action induces the activation of signaling pathways such as the Jak-Stat pathway [56, 57], important during the cytokine mediated immune response and during regulation of proliferation, migration and apoptosis. Recent studies indicate a non-canonical role for some elements of this pathway that indicate their participation in an increase in tumorigenesis [56].

Interestingly, the downregulated proteins identified are all of them susceptible to the modification by ubiquitin protein C (UBC); this modification confers several effects on the target protein. Examples of this are protein activation or inactivation, protein-protein interaction, vesicular trafficking and degradation through proteasome [58].

These changes reflect a general response towards EOC ascites from SKOV-3 ovarian cancer cell line. Some of these changes occur as a response of malignant cells to survive and avoid the damage induced by the host immune response, whereas other changes obey to build a microenvironment where signals of proliferation or signals that promote invasion and metastases towards other organs predominate to guarantee their survival and domination [59, 60].

This work reveals the modulation of different proteins in the SKOV-3 cell line under the effect of EOC ascites. However, it is necessary to continue the characterization of this effect using a greater number of ascites. In addition, a future analysis should consider the effect of this fluid on multicellular spheroids, which are frequently found in ascites and are considered responsible for the development of micro implants in the abdominopelvic cavity.

## Conclusion

The analysis of the proteome of malignant cells under the effect of ascites reflects the activation of very diverse biological processes. Therefore, we studied further some proteins that reveal the participation of specific important processes such as cell cycle, vesicular transport, and evasion of the immune response, degradation and modification of the extracellular matrix, and resistance to chemotherapeutic agents, all of them of high relevance for the aggressive behavior of malignant cells.

Thus, this work may represent a source of information which has the potential to be evaluated for the design of therapies directed against these malignant cells that reside within ovarian cancer ascites.

## Abbreviations

2-DE: Two-dimensional gel electrophoresis
CS: Clinical stages
EOC: Epithelial ovarian cancer
GOTERM: Gene Ontology Terms
PPI: Protein-protein interaction
